# Effect of Multimorbidity on Health-Related Quality of Life in Adults Aged 55 Years or Older: Results from the SU.VI.MAX 2 Cohort

**DOI:** 10.1371/journal.pone.0169282

**Published:** 2016-12-29

**Authors:** Valentin Walker, Christine Perret-Guillaume, Emmanuelle Kesse-Guyot, Nelly Agrinier, Serge Hercberg, Pilar Galan, Karen E. Assmann, Serge Briançon, Christine Rotonda

**Affiliations:** 1 EA4360 APEMAC, University of Lorraine, University Paris Descartes, Nancy, France; 2 Department of Gerontology, CHRU de Brabois, Nancy, France; 3 Equipe de Recherche en Epidémiologie Nutritionnelle, Centre d’Epidémiologie et Statistiques Paris Cité, Université Paris 13, Inserm (U1153), Inra (U1125), COMUE Sorbonne-Paris-Cité, Bobigny, France; 4 CIC-1433 Epidémiologie Clinique, Inserm, CHRU Nancy, Nancy, France; 5 Département de Santé Publique, Hôpital Avicenne, Bobigny, France; Iranian Institute for Health Sciences Research, ISLAMIC REPUBLIC OF IRAN

## Abstract

**Introduction:**

Multimorbid chronic diseases are usually considered separately in trials. Here, we aimed to describe overall multimorbidity patterns in adults aged 55 years or older and assess their effect on health-related quality of life (HRQoL).

**Methods:**

We used data for 5,647 participants included in the SUpplémentation en VItamines et Minéraux AntioXydants 2 (SU.VI.MAX 2) population-based trial. HRQoL was assessed by the French versions of the Medical Outcome Study Short Form 36 and the Duke Health Profile. An exploratory factor analysis was used to determine multimorbidity patterns, and a multimorbidity score for each resulting pattern was calculated. Adjusted multiple linear regression was used to examine the association between the identified multimorbidity and HRQoL scores by gender and for each age group (55–59, 60–64, 65–69, ≥ 70 years).

**Results:**

More than 63% of the sample reported two or more chronic conditions (from 55.8% for those 55–59 years to 74.4% for those ≥ 70 years). Multimorbidity was more common among women than men (67.3% vs 60%). Two different multimorbidity patterns were identified. Pattern A was represented mainly by mental illness and bone impairments. Pattern B was represented mainly by cardiovascular and metabolic disorders. After adjusting for covariates, a high pattern A score was associated with reduced HRQoL for the physical and mental components of each HRQoL questionnaire, and a high pattern B score was associated with reduced HRQoL for only the physical component of each questionnaire. These multimorbidity scores affected HRQoL differently by age group.

**Conclusion:**

Our study used a novel methodological approach to account for multimorbidity patterns in determining the link with chronic conditions. These multimorbidity scores (counted and weighted) can be used in clinical research to control for the effect of multimorbidity on patients’ HRQoL and may be useful for clinical practice.

**Clinical Trial Registration:**

Clinicaltrial.gov (number NCT00272428).

## Introduction

The European population is the most aged in the world, with 24% of the population 60 years or older. It is projected to remain the most aged population in the coming decades, with 34% of the population projected to be 60 years or older in 2050 [1]. This overall aging of the population is accompanied by a substantial increase in prevalence of chronic conditions. Two thirds of older adults in Europe who have reached retirement age have at least two chronic conditions [2,3].

This co-existence of multiple chronic conditions, defined as “multimorbidity”, is a common phenomenon in older people, and its occurrence increases with age [4]. Multimorbid chronic diseases are associated with increased rate of mortality and disability, reduced function levels, increased polypharmacy, poor health-related quality of life (HRQoL) and more health care utilization (costs, number of physician visits, length of hospital stay) [5–7]. In this context, health care should aim to increase the life span cost-efficiently while maintaining HRQoL and the ability to perform activities of daily life [8,9]. Most studies have shown impaired HRQoL by gender with the presence of many chronic diseases or with aging in older people [10–13].

When studying the impact of morbidities on HRQoL, morbidities are usually considered separately [2]. Most treatment plans and clinical guidelines target single diseases [14], but an effective intervention for one disease could be less effective or deleterious with the presence of coexisting conditions [15]. Regarding associations between morbidities, patterns of morbidities can be established. However, few studies are identifying patterns and potential factors underlying such associations [16–20]. The interest of these patterns is to consider the interrelations or the cumulative effect between different morbidities. Methodological approaches that consider such patterns that are well-adapted to the respective study populations are needed, as is the need to understand the patterns of disease combinations and their complexity. The identification and standardization of patterns of multimorbidity might help in organizing specific treatment strategies and system-wide initiatives to improve the care of people with various types and degrees of multimorbidity. However, more evidence on multimorbidity patterns is required.

We aimed to describe the multimorbidity patterns in adults aged 55 years or older by using national French data from the Supplémentation en VItamines et Minéraux AntioXydants 2 (SU.VI.MAX 2) study. We also aimed to assess the association between multimorbidity patterns and HRQoL among older people overall and by age and gender.

## Materials and Methods

### Study design

Our study is based on the data from SU.VI.MAX 2 study, which is an additional observational follow-up study, organized 5 years after the end of the initial SU.VI.MAX trial. The initial SU.VI.MAX trial was a randomized, double-blind, placebo-controlled primary prevention trial assessing the efficacy of a daily antioxidant supplementation in the incidence of cardiovascular disease and cancer. Eligibility criteria of SU.VI.MAX participants were described in previous publication [21–23]. This initial trial was launched in 1994–95 and had a planned follow-up of 8 years (until 2002).

The SU.VI.MAX and SU.VI.MAX 2 studies were approved by the Ethics Committee for Studies with Human Subjects of the Paris-Cochin Hospital (CCPPRB nos. 706 and 2364, respectively) and the Commission Nationale Informatique et Liberté (CNIL nos. 334641 and 907094, respectively). Written informed consent was obtained from all participants. Clinical Trial Registration at clinicaltrial.gov (number NCT00272428).

### Population and sampling

**Fig 1:**
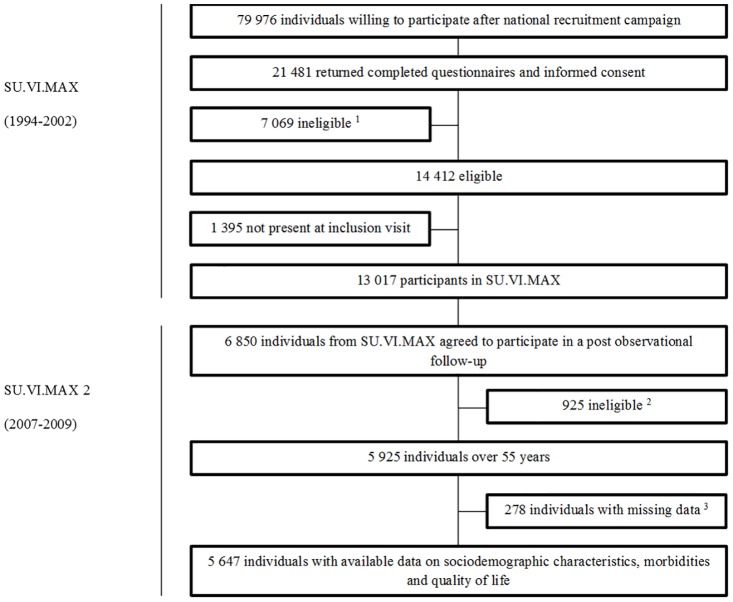
Enrollment and flow of participants in the Supplémentation en Vitamines et Minéraux Antioxydants (SU.VI.MAX) study and follow-up (SU.VI.MAX 2). ^1^ Doesn’t met eligibility criteria. ^2^ Under 55 years. ^3^ Missing data about sociodemographic characteristics, morbidities and quality of life.

The SU.VI.MAX 2 participants were recruited through a postal campaign organized in 2007–2009 among all SU.VI.MAX participants. From the full initial SU.VI.MAX cohort (N = 13,017), 6,850 participants were agreed to participate in the SU.VI.MAX 2 study, and 5,925 participants were older than 55 years at enrollment and received a geriatric assessment [24,25]. Sociodemographic characteristics data, morbidities data and quality of life data were available for 5,647 aged 55 years or older for inclusion in the present analyses (Fig 1).

### Data collection

#### Sociodemographic and comorbidities assessment

Information on gender, date of birth, weight, size, employment status, education level, familial status, employment status and smoking status was collected.

In SU.VI.MAX 2, subjects reported their comorbidities by a self-administered questionnaire. Several chronic conditions were identified in 12 categories: cardiovascular diseases or other cardiovascular impairments; cancers; respiratory diseases or impairments; diseases or impairments related to the ears, nose, or throat; digestive diseases or impairments of digestive function; diseases or impairments related to bones and joints; urinary/genital diseases or impairments; endocrine or metabolic diseases; eye-related diseases or impairments; neurological/psychiatric diseases or impairments; infectious diseases; and other health problems. The year of diagnosis was collected, as was the presence of a medication or actual treatment for the condition. On the basis of the potential relevance of conditions for a participant’s overall health status, one of the authors (CPG), a geriatrician, selected 19 chronic conditions: hypertension, heart failure, arrhythmias and palpitations, ischemic cardiovascular/vascular impairments, respiratory impairments, hearing impairments, ear, nose and throat impairments, digestive impairments, vertebral diseases, osteoporosis, arthritis and rheumatism, adenoma or prostatic hyperplasia, thyroid disease, diabetes, vision impairments, anxiety/depression, sleeping troubles, memory impairments and cancer. Multimorbidity was defined as having at least 2 of these 19 chronic conditions.

#### Self-reported outcomes: HRQoL

HRQoL was assessed (2007–2009) at enrollment by using the French version of the Medical Outcome Study Short Form 36 (SF-36) [26] and the French version of the Duke Health Profile (Duke) [27]. For each questionnaire, two main dimensions were retained: the physical component (SF-36: physical component summary [PCS]; Duke: Phys) and the mental component (SF-36: mental component summary; Duke: Ment). The scores were linearized from 0 to 100 to compare scores between the questionnaires: (0: worst HRQoL, 100: best HRQoL).

#### Statistical analysis

We performed a descriptive analysis, reporting unweighted frequencies, proportions, and means (SD) by gender and age groups (55–59, 60–64, 65–69 and ≥ 70 years).

We performed an exploratory factor analysis of the 19 morbidities to determine multimorbidity patterns. We identified the tendencies of diseases to co-occur by selecting sets of variables with potentially common underlying causal factors. Factor analysis was used with a tetrachoric correlation matrix because conditions were expressed as binary variables [28]. The extraction of the multimorbidity patterns involved the principal factor method, and the number of factors to extract was determined by the scree-test [29], with minimal eigenvalue of 1.0 (Kaiser criterion). A condition with loading factor > 0.25 had greater importance in a pattern, which indicates a stronger association [30]. The Kaiser-Meyer-Olkin (KMO) method was used to estimate the adequacy of the data for our model on factor analysis. This parameter takes values between 0 and 1, which, with a greater goodness of fit, are close to 1. Cumulative variance of the sample was determined to describe the variance of the diagnostic data explained by the patterns. An oblique rotation (Oblimin) was applied to correlate factors with one another to obtain a better interpretation of the analysis factor. The results of this analysis could be interpreted as multimorbidity patterns (i.e., diseases that are non-randomly associated with each other).

For each participant, a multimorbidity score was calculated for every identified pattern. These individual scores corresponded to the sum of each loading factor from the factor analysis multiplied by the presence (= 1) or absence (= 0) of each condition. The mean of each multimorbidity score was calculated for every pattern. The higher the multimorbidity score, the greater the number and association of multimorbidities.

We hypothesized that multimorbidity scores’ impact on HRQoL was different according age and gender. Significant interactions between age groups and multimorbidity scores and between gender and multimorbidity scores confirmed this hypothesis. So multiple linear regression used to examine the association between multimorbidity and HRQoL scores (PCS and Phys; MCS and Ment) were realized for each age group and by gender. Models were adjusted for others sociodemographic variables which p<0.2 in bivariate analyses. Analyses involved use of SAS 9.4. Two-sided p < 0.001 was considered statistically significant (after Bonferroni correction).

## Results

### Sample

Among the 5647 adults aged 55 years or older (51.5% women), the mean (SD) age was 63.2 years (4.9); 71.2% were retired and 66% declared a good general health status (data not shown). For participants 60–64 years old, 12.8% of women were working as compared with 18.7% of men (Table 1). For participants 55–59 years old, 36.0% of men were retired as compared with 23.6% of women. Among those ≥ 70 years old, 46.3% of men had a university education and 10.9% were single as compared with 36.9% and 35.3% of women, respectively. Among those 65–69 years, 29.6% of men never smoked as compared with 64.1% of women.

**Table 1:** Description of participants aged 55 years or older in the SU.VI.MAX 2 study by gender and age group.

	TOTAL	MALE (n = 2738, 48.5%)					FEMALE (n = 2909, 51.5%)				
		55–59 years	60–64 years	65–69 years	≥70 years	Total	55–59 years	60–64 years	65–69 years	≥70 years	Total
**Sample**	5647 (100)	497 (18.2)	1026 (37.5)	748 (27.3)	467 (17.1)	2738 (100)	978 (33.6)	976 (33.6)	635 (21.8)	320 (11.0)	2909 (100)
**Age**	63.2 (4.9)	58.0 (1.1)	61.8 (1.5)	67.0 (1.4)	71.3 (1.3)	64.2 (4.7)	57.2 (1.4)	61.7 (1.4)	66.9 (1.4)	71.3 1.2)	62.4 (4.9)
**BMI**	25.7 (4.1)	26.2 (3.4)	26.6 (3.8)	26.5 (3.4)	26.5 (3.3)	26.5 (3.6)	24.6 (4.5)	25.0 (4.4)	25.2 (4.3)	26.0 (5.0)	25.0 (4.5)
**Employment**											
Working	1188 (21.0)	281 (56.5)	192 (18.7)	20 (2.7)	5 (1.1)	498 (18.2)	544 (55.6)	125 (12.8)	17 (2.7)	4 (1.3)	690 (23.7)
Unemployed	436 (7.7)	37 (7.4)	28 (2.7)	4 (0.5)	3 (0.6)	72 (2.6)	203 (20.8)	128 (13.1)	24 (3.8)	9 (2.8)	364 (12.5)
Retired	4023 (71.2)	179 (36.0)	806 (78.6)	724 (96.8)	459 (98.3)	2168 (79.2)	231 (23.6)	723 (74.1)	594 (93.5)	307 (96.0)	1855 (63.8)
**Education**											
No education	36 (0.6)	2 (0.4)	0 (0)	7 (0.9)	7 (1.5)	16 (0.6)	3 (0.3)	4 (0.4)	7 (1.1)	6 (1.9)	20 (0.7)
Primary	375 (6.6)	18 (3.6)	56 (5.5)	68 (9.1)	50 (10.7)	192 (7.0)	41 (4.2)	57 (5.8)	50 (7.9)	35 (10.9)	183 (6.3)
Secondary	2387 (42.3)	185 (37.2)	387 (37.7)	298 (39.8)	194 (41.5)	1064 (38.9)	410 (41.9)	432 (44.3)	320 (50.4)	161 (50.31)	1323 (45.5)
University	2849 (50.5)	292 (58.8)	583 (56.8)	375 (50.1)	216 (46.3)	1466 (53.5)	524 (53.6)	483 (49.5)	258 (40.6)	118 (36.9)	1383 (47.5)
**Family status**											
Single	955 (16.9)	52 (10.5)	101 (9.8)	90 (12.0)	51 (10.9)	294 (10.7)	189 (19.3)	196 (20.1)	163 (25.7)	113 (35.3)	661 (22.7)
Couple	4549 (80.6)	435 (87.5)	911 (88.8)	645 (86.2)	409 (87.6)	2400 (87.7)	752 (76.9)	743 (76.1)	457 (72.0)	197 (61.6)	2149 (73.9)
Living with another person	143 (2.5)	10 (2.0)	14 (1.4)	13 (1.7)	7 (1.5)	44 (1.6)	37 (3.8)	37 (3.8)	15 (2.4)	10 (3.1)	99 (3.4)
**Smoking status**											
Never-smokers	2550 (45.2)	163 (32.8)	335 (32.7)	221 (29.6)	127 (27.2)	846 (30.9)	512 (52.4)	573 (58.7)	407 (64.1)	212 (66.3)	1704 (58.6)
Former smokers	2761 (48.9)	298 (60.0)	616 (60.0)	475 (63.5)	324 (69.4)	1713 (62.6)	399 (40.8)	341 (34.9)	209 (32.9)	99 (30.9)	1048 (36.0)
Current smokers	336 (5.9)	36 (7.2)	75 (7.3)	52 (7.0)	16 (3.4)	179 (6.5)	67 (6.9)	62 (6.4)	19 (3.0)	9 (2.8)	157 (5.4)

Data are mean (SD) or unweight frequencies (%). BMI, body mass index.

### Chronic conditions and prevalence of multimorbidity

More than 87% of the participants reported having at least one chronic condition, and more than 63% had at least two chronic conditions. The most frequent chronic diseases were arthritis/rheumatism, vision impairments and hypertension, with an overall prevalence of 41.0%, 40.5% and 23.9%, respectively (Table 2). For women, the most represented chronic conditions were arthritis and rheumatism (47.7%), vision impairments (43.3%), anxiety/depression (24.6%) and sleeping troubles (25.3%) (Table 2). For men, the most represented chronic conditions were vision impairments (37.5%), arthritis and rheumatism (33.8%) and hypertension (27.4%). The proportion of multimorbidity was greater for women than men (67.3% and 60.0%, respectively). For men and women, presence of an increasing number of chronic conditions increased with increasing age (Table 2). The proportion of each morbidity increased with age for men (hypertension– 55–59 years: 20.3%, ≥ 70 years: 32.6%; arthritis and rheumatism– 55–59 years: 22.7%, ≥70 years: 43.5%) and women (hypertension– 55–59 years: 16.9%, ≥70 years: 26.6%; arthritis and rheumatism– 55–59 years: 38.7%, ≥70 years: 60.9%), except for 4 conditions for which no increase was observed (hearing impairment, nose and throat impairments, digestive impairments, osteoporosis, sleeping troubles). The prevalence of only anxiety/depression tended to decreased with age for men (55–59 years: 15.3%, ≥70 years: 9.9%) and women (55–59 years: 26.9%, ≥70 years: 22.2%), but this decrease was greater for women. Other chronic conditions, such as sleeping troubles, had a higher frequency among women than men regardless of age group [men (55–59 years: 13.1%, ≥70 years: 14.6%) and women (55–59 years: 26.6%, ≥70 years: 27.2%)].

**Table 2:** Prevalence of chronic diseases in adults aged 55 years or older in the SU.VI.MAX 2 study by gender and age group.

	TOTAL	MALE (n = 2738, 48.5%)					FEMALE (n = 2909, 51.5%)				
		55–59 years	60–64 years	65–69 years	≥ 70 years	Total	55–59 years	60–64 years	65–69 years	≥70 years	Total
**Hypertension**	1347 (23.9)	101 (20.3)	253 (24.7)	243 (32.5)	152 (32.6)	**749 (27.4)**	165 (16.9)	196 (20.1)	152 (23.9)	85 (26.6)	598 (20.6)
**Heart failure**	81 (1.4)	5 (1.0)	11 (1.1)	18 (2.4)	19 (4.1)	53 (1.9)	9 (0.9)	5 (0.5)	8 (1.3)	6 (1.9)	28 (1.0)
**Arrhythmias and palpitations**	276 (4.9)	13 (2.6)	40 (3.9)	51 (6.8)	40 (8.6)	144 (5.3)	31 (3.2)	38 (3.9)	43 (6.8)	20 (6.3)	132 (4.5)
**Ischemic cardiovascular/vascular impairments**	347 (6.1)	21 (4.2)	72 (7.0)	87 (11.6)	67 (14.4)	247 (9.0)	24 (2.5)	20 (2.1)	35 (5.5)	21 (6.6)	100 (3.4)
**Respiratory impairments**	292 (5.2)	31 (6.2)	43 (4.2)	38 (5.1)	26 (5.6)	138 (5.0)	44 (4.5)	39 (4.0)	40 (6.3)	31 (9.7)	154 (5.3)
**Hearing impairments**	913 (16.2)	68 (13.7)	172 (16.8)	168 (22.5)	134 (28.7)	542 (19.8)	99 (10.1)	107 (11.0)	99 (15.6)	66 (20.6)	371 (12.8)
**Ear, nose and throat impairments**	162 (2.9)	12 (2.4)	25 (2.4)	30 (4.0)	6 (1.3)	73 (2.7)	28 (2.9)	30 (3.1)	19 (3.0)	12 (3.8)	89 (3.1)
**Digestive impairments**	526 (9.3)	34 (6.8)	84 (8.2)	49 (6.6)	44 (9.4)	211 (7.7)	93 (9.5)	101 (10.4)	78 (12.3)	43 (13.4)	315 (10.8)
**Vertebral diseases**	430 (7.6)	30 (6.0)	50 (4.9)	42 (5.6)	37 (7.9)	159 (5.8)	75 (7.7)	84 (8.6)	70 (11.0)	42 (13.1)	271 (9.3)
**Osteoporosis**	477 (8.5)	8 (1.6)	12 (1.2)	14 (1.9)	5 (1.1)	39 (1.4)	120 (12.3)	152 (15.6)	107 (16.9)	59 (18.4)	438 (15.1)
**Arthritis and rheumatism**	2313 (41.0)	113 (22.7)	319 (31.1)	291 (38.9)	203 (43.5)	**926 (33.8)**	378 (38.7)	472 (48.4)	342 (53.9)	195 (60.9)	**1387 (47.7)**
**Adenoma or prostatic hyperplasia**	325 (5.8)	26 (5.2)	111 (10.8)	116 (15.5)	72 (15.4)	325 (11.9)	0 (0.0)	0 (0.0)	0 (0.0)	0 (0.0)	0 (0.0)
**Thyroid disease**	454 (8.0)	6 (1.2)	32 (3.1)	22 (2.9)	14 (3.0)	74 (2.7)	133 (13.6)	117 (12.0)	74 (11.7)	56 (17.5)	380 (13.1)
**Diabetes**	161 (2.9)	15 (3.0)	27 (2.6)	49 (6.6)	24 (5.1)	115 (4.2)	9 (0.9)	17 (1.7)	13 (2.1)	7 (2.2)	46 (1.6)
**Vision impairments**	2286 (40.5)	152 (30.6)	371 (36.2)	285 (38.1)	219 (46.9)	**1027 (37.5)**	397 (40.6)	400 (41.0)	282 (44.4)	180 (56.3)	**1259 (43.3)**
**Anxiety / depression**	1058 (18.7)	76 (15.3)	136 (13.3)	84 (11.2)	46 (9.9)	342 (12.5)	263 (26.9)	247 (25.3)	135 (21.3)	71 (22.2)	**716 (24.6)**
**Sleeping troubles**	1175 (20.8)	65 (13.1)	185 (18.0)	120 (16.0)	68 (14.6)	438 (16.0)	260 (26.6)	237 (24.3)	153 (24.1)	87 (27.2)	**737 (25.3)**
**Memory impairments**	334 (5.9)	13 (2.6)	39 (3.8)	39 (5.2)	30 (6.4)	121 (4.4)	48 (4.9)	62 (6.4)	64 (10.1)	39 (12.2)	213 (7.3)
**Cancer**	309 (5.5)	19 (3.8)	58 (5.7)	63 (8.4)	48 (10.3)	188 (6.9)	25 (2.6)	44 (4.5)	29 (4.6)	23 (7.2)	121 (4.2)
**Number of chronic conditions**											
0	721 (12.8)	111 (22.3)	174 (17.0)	86 (11.5)	44 (9.4)	415 (15.2)	141 (14.4)	101 (10.4)	48 (7.6)	16 (5.0)	306 (10.5)
1	1325 (23.5)	165 (33.2)	270 (26.3)	168 (22.5)	78 (16.7)	681 (24.9)	235 (24.0)	224 (23.0)	125 (19.7)	60 (18.8)	644 (22.1)
2	1315 (23.3)	109 (21.9)	256 (25.0)	162 (21.7)	112 (24.0)	639 (23.3)	230 (23.5)	247 (25.3)	144 (22.7)	55 (17.2)	676 (23.2)
3	1007 (17.8)	63(12.7)	174 (17.0)	161 (21.5)	100 (21.4)	498 (18.2)	163 (16.7)	175 (17.9)	119 (18.7)	52 (16.3)	509 (17.5)
4+	1279 (22.7)	49 (9.9)	152 (14.8)	171 (22.9)	133 (28.5)	505 (18.4)	209 (21.4)	229 (23.5)	199 (31.3)	137 (42.8)	774 (26.6)
**Multimorbidity**	3601 (63.8)	221 (44.5)	582 (56.7)	494 (66.0)	345 (73.9)	1642 (60.0)	602 (61.6)	651 (66.7)	462 (72.8)	244 (76.3)	1959 (67.3)

Data are unweighted frequencies (n) and proportions (%).

### HRQoL

HRQoL scores are in Table 3.

**Table 3:** Outcomes related to mental health, physical health and health-related quality of life (HRQoL) by gender and age groups.

	MALE					FEMALE				
	55–59 years	60–64 years	65–69 years	≥ 70 years	Total	55–59 years	60–64 years	65–69 years	≥ 70 years	Total
**SF-36 (PCS)**	52.2 (6.1)	50.8 (6.5)	50.1 (6.6)	49.3 (6.6)	50.6 (6.5)	50.9 (7.1)	50.4 (7.1)	48.3 (8.0)	46.9 (8.0)	49.7 (7.5)
**SF-36 (MCS)**	50.9 (9.5)	52.6 (7.9)	52.5 (7.8)	52.3 (7.5)	52.2 (8.2)	48.6 (9.6)	49.4 (9.3)	49.8 (9.5)	49.6 (9.9)	49.2 (9.5)
**DUKE (Phys)**	81.2 (16.9)	80.5 (17.0)	78.7 (17.0)	76.3 (17.9)	79.4 (17.2)	71.9 (19.0)	70.8 (19.2)	68.7 (19.6)	65.7 (21.0)	70.2 (19.5)
**DUKE (Ment)**	80.7 (19.1)	83.8 (16.9)	83.1 (16.4)	83.9 (16.7)	83.1 (17.2)	73.5 (20.5)	75.0 (20.0)	75.9 (18.9)	77.0 (18.8)	75.0 (19.9)

Data are mean (SD). SF-36, Medical Outcomes Study Short Form 36. DUKE, Duke Health Profile.

PCS, physical component summary; MCS, mental component summary; Phys, physical health; Ment, mental health.

### Multimorbidity patterns

We identified two multimorbidity patterns according to the eigenvalues of the factor analysis and the results of the scree-test, which explained 62.2% of the total variance (Table 4). The KMO value was 0.61, which was considered acceptable adequacy. Three conditions were less associated with the two patterns, A and B, because of factor loading < 0.25 (hearing impairment, vision impairments and cancer). With the threshold of 0.25, the variable respiratory impairments were correlated with both patterns (factor loading 0.26 and 0.30).

**Table 4:** Explanatory factor analysis for two multimorbidity patterns, A and B, for each condition.

Conditions	Pattern A	Pattern B
**Hypertension**	-0.01	**0.50**
**Heart failure**	0.10	**0.57**
**Arrhythmias and palpitations**	0.16	**0.32**
**Ischemic cardiovascular impairments**	-0.01	**0.52**
**Respiratory impairments**	**0.26**	**0.30**
**Hearing impairment**	0.16	0.17
**Ear, nose and throat impairments**	**0.39**	0.01
**Digestive impairments**	**0.34**	0.10
**Vertebral diseases**	**0.36**	0.10
**Osteoporosis**	**0.35**	-0.32
**Arthritis and rheumatism**	**0.44**	-0.04
**Adenoma or prostatic hyperplasia**	-0.15	**0.44**
**Thyroid disease**	**0.29**	-0.05
**Diabetes**	-0.14	**0.62**
**Vision impairments**	0.22	0.15
**Anxiety / depression**	**0.62**	-0.11
**Sleeping troubles**	**0.68**	-0.02
**Memory impairments**	**0.65**	0
**Cancer**	0.06	0.10

Pattern scores >0.25 are highlighted.

Kaiser-Meyer-Olkin (KMO) value: 0.613.

Pattern A included respiratory impairments; ear, nose and throat impairments; digestive impairments; vertebral diseases; osteoporosis; arthritis and rheumatism; thyroid disease; anxiety/depression; sleeping troubles and memory impairments.

Pattern B included hypertension, heart failure, arrhythmias and palpitations, ischemic cardiovascular/vascular impairments, respiratory impairments, adenoma or prostatic hyperplasia and diabetes.

### Multimorbidity score

We established a pattern A and B score for each participant corresponding to the overall impact of these chronic conditions (mean [SD] score 0.73 [0.69] and 0.27 [0.40], respectively) (data not shown). The mean pattern A score for men was 0.55 [range: -0.29; 3.63] and for women 0.89 [range: -0.15; 4.31]. The mean pattern B score for men was 0.38 [range: -0.46; 2.70] and for women 0.17 [range: -0.54; 3.24].

Mean pattern A scores for men were 0.46, 0.54, 0.57 and 0.63 for ages 55–59, 60–64, 65–69 and ≥70, respectively, and for women 0.83, 0.87, 0.94 and 1.09, respectively. Mean pattern B scores for men were 0.25, 0.32, 0.46 and 0.52, respectively, and for women 0.13, 0.15, 0.22 and 0.27, respectively.

### Association of multimorbidity scores and HRQoL

Tables 5 and 6 show the linear regression analysis of the association of multimorbidity and HRQoL scores by age groups for men and women, adjusted for covariates (BMI, professional status, education level, family status and smoking status). For men, after adjusting for covariates, increased pattern A score was associated with reduced HRQoL score for all four dimensions studied whatever the age group (Table 5). Increased adjusted pattern B score was associated with only Duke Phys dimension (60–64 years: -5.3; p<0.0001) and the association tended to decrease with age (55–59 years: -5.7; 60–64 years: -5.3; 65–69 years: -3.4; ≥70 years: -3.5).

**Table 5:** Impact of multimorbidity on HRQoL for males.

		MALE							
		55–59 years		60–64 years		65–69 years		≥ 70 years	
		Beta	P	Beta	P	Beta	P	Beta	P
**SF-36 (PCS)**	**Intercept**	61.0	**<0.0001**	64.5	**<0.0001**	58.2	**<0.0001**	56.8	**<0.0001**
	**Pattern A Score**	-2.29	**<0.0001**	-3.49	**<0.0001**	-2.39	**<0.0001**	-2.95	**<0.0001**
	**Pattern B Score**	-3.29	**<0.0001**	-3.36	**<0.0001**	-2.39	**<0.0001**	-1.95	**0.0023**
	Adjusted Pattern A Score*	-1.9	**<0.0001**	-3.0	**<0.0001**	-2.1	**<0.0001**	-2.7	**<0.0001**
	Adjusted Pattern B Score*	-2.3	**0.0027**	-1.6	**0.0021**	-1.8	**0.0005**	-1.3	**0.0391**
**SF-36 (MCS)**	**Intercept**	53.4	**<0.0001**	54.3	**<0.0001**	56.3	**<0.0001**	55.1	**<0.0001**
	**Pattern A Score**	-5.39	**<0.0001**	-4.07	**<0.0001**	-3.13	**<0.0001**	-4.38	**<0.0001**
	**Pattern B Score**	-0.96	0.4244	-1.35	**0.0380**	-0.91	0.1307	-0.92	0.21
	Adjusted Pattern A Score*	-5.4	**<0.0001**	-4.0	**<0.0001**	-3.1	**<0.0001**	-4.4	**<0.0001**
	Adjusted Pattern B Score*	/		-0.6	0.3607	-0.5	0.4128	/	
**Duke (Phys)**	**Intercept**	112.4	**<0.0001**	114.3	**<0.0001**	105.4	**<0.0001**	106.9	**<0.0001**
	**Pattern A Score**	-10.6	**<0.0001**	-12.9	**<0.0001**	-10.9	**<0.0001**	-13.7	**<0.0001**
	**Pattern B Score**	-8.13	**0.0001**	-10.5	**<0.0001**	-5.81	**<0.0001**	-6.35	**0.0003**
	Adjusted Pattern A Score*	-9.8	**<0.0001**	-11.7	**<0.0001**	-10.3	**<0.0001**	-12.8	**<0.0001**
	Adjusted Pattern B Score*	-5.7	**0.0047**	-5.3	**<0.0001**	-3.4	**0.0057**	-3.5	**0.0280**
**Duke (Ment)**	**Intercept**	92.8	**<0.0001**	87.3	**<0.0001**	88.4	**<0.0001**	86.3	**<0.0001**
	**Pattern A Score**	-11.8	**<0.0001**	-10.5	**<0.0001**	-7.13	**<0.0001**	-10.9	**<0.0001**
	**Pattern B Score**	-1.91	0.4262	-3.69	**0.0077**	-3.81	**0.0024**	-2.03	0.2154
	Adjusted Pattern A score*	-11.5	**<0.0001**	-10.3	**<0.0001**	-6.9	**<0.0001**	-10.9	**<0.0001**
	Adjusted Pattern B Score*	/		-1.6	0.2276	-2.9	**0.0177**	/	

* Linear regression model for each age class was adjusted for body mass index (BMI), professional status, education level, family status and smoking status.

**Table 6:** Impact of multimorbidity on HRQoL for females.

		FEMALE							
		55–59 years		60–64 years		65–69 years		≥70 years	
		Beta	P	Beta	P	Beta	P	Beta	P
**SF-36 (PCS)**	**Intercept**	57.4	**<0.0001**	61.5	**<0.0001**	64.4	**<0.0001**	60.0	**<0.0001**
	**Pattern A Score**	-3.32	**<0.0001**	-2.9	**<0.0001**	-2.91	**<0.0001**	-2.66	**<0.0001**
	**Pattern B Score**	-5.04	**<0.0001**	-3.72	**<0.0001**	-1.85	**0.0343**	-5.28	**<0.0001**
	Adjusted Pattern A score*	-3.1	**<0.0001**	-2.8	**<0.0001**	-2.8	**<0.0001**	-2.3	**<0.0001**
	Adjusted Pattern B Score*	-3.9	**<0.0001**	-2.8	**<0.0001**	-0.9	0.2957	-2.3	**0.0326**
**SF-36 (MCS)**	**Intercept**	47.4	**<0.0001**	40.9	**<0.0001**	47.6	**<0.0001**	54.5	**<0.0001**
	**Pattern A Score**	-3.79	**<0.0001**	-4.22	**<0.0001**	-3.85	**<0.0001**	-4.67	**<0.0001**
	**Pattern B Score**	2.32	**0.0227**	-0.25	0.7819	1.25	0.2300	-2.18	0.0992
	Adjusted Pattern A score*	-3.9	**<0.0001**	-4.0	**<0.0001**	-3.8	**<0.0001**	-4.6	**<0.0001**
	Adjusted Pattern B Score*	1.6	0.1070	/		/		-0.5	0.7096
**Duke (Phys)**	**Intercept**	91.3	**<0.0001**	103.7	**<0.0001**	103.9	**<0.0001**	105.0	**<0.0001**
	**Pattern A Score**	-11.1	**<0.0001**	-11.6	**<0.0001**	-10.5	**<0.0001**	-10.5	**<0.0001**
	**Pattern B Score**	-11.6	**<0.0001**	-7.34	**<0.0001**	-6.33	**0.0030**	-17.2	**<0.0001**
	Adjusted Pattern A score*	-10.3	**<0.0001**	-11.2	**<0.0001**	-10.1	**<0.0001**	-9.0	**<0.0001**
	Adjusted Pattern B Score*	-7.8	**<0.0001**	-4.9	**0.0034**	-3.5	0.0704	-9.0	**0.0006**
**Duke (Ment)**	**Intercept**	80.3	**<0.0001**	73.2	**<0.0001**	96.9	**<0.0001**	87.2	**<0.0001**
	**Pattern A Score**	-9.18	**<0.0001**	-10.3	**<0.0001**	-9.24	**<0.0001**	-9.36	**<0.0001**
	**Pattern B Score**	1.28	0.5574	-2.22	0.2531	0.65	0.7526	-2.83	0.2621
	Adjusted Pattern A score*	-9.1	**<0.0001**	-9.9	**<0.0001**	-9.0	**<0.0001**	-9.4	**<0.0001**
	Adjusted Pattern B Score*	/		/		/		/	

* Linear regression model for each age class was adjusted for BMI, professional status, education level, family status and smoking status.

The strongest association was between an increase in both morbidity scores and reduced HRQoL measured by the DUKE questionnaire. This association remained strong after adjusting for covariates for the pattern A but not pattern B score (e.g., DUKE Ment, 55–59 years: adjusted pattern A score: -11.5, p<0.0001; adjusted pattern B score: not significant).

For women, the same results were found (Table 6). Increased adjusted pattern A score was associated with reduced HRQoL score for all four dimensions studied whatever the age group. Increased adjusted pattern B score was associated with some age groups for the SF-36 PCS (55–59 years: -3.9, p<0.0001; 60–64 years: -2.8, p<0.0001) and the DUKE Phys (55–59 years: -7.8, p<0.0001; ≥ 70 years: -9.0, p = 0.0006). This association tended to decrease for the SF-36 PCS (55–59 years: -3.9; 60–64 years, -2.8; ≥70 years: -2.3), whereas this decrease was maximal for the extreme age groups for the DUKE Phys (55–59 years: -7.8; ≤70 years: -9.0).

## Discussion

In a sample of 5647 subjects aged 55 years or older, an exploratory factor analysis allowed for identifying two multimorbidity patterns: A and B. On exploring the cross-sectional association of individual multimorbidity and HRQoL scores (assessed by the SF-36 and DUKE questionnaires), pattern A explained more of the HRQoL score decrease than pattern B, with mean multimorbidity scores of 0.73 and 0.27 for the two patterns, respectively. HRQoL was better for men in general, and mental dimension scores tended to increase with age and physical dimension decreased.

The characteristics of the patterns we extracted were similar to those observed previously [31]. The most important conditions in pattern A concerned mental illness and bone impairments. Anxiety is associated with depression problems [32,33]. These conditions could induce sleep disorders, thereby causing some potential memory impairments that may explain the mental part of this pattern. Nevertheless, depression problems may increase with increasing pain and significant limitations in movement. Regarding the relationship between mental disorders and bone disorders, the World Mental Health Surveys, conducted across 17 countries, found greater risk of developing mood disorders and anxiety with presence of osteoarthritis [34]. Being in constant pain, with limited movement, may lead to doubts about the ability to be autonomous and thus a negative self-image inducing anxiety and depressive disorders.

Pattern B was found in previous studies [31]. Its composition is largely associated with cardiovascular and metabolic disorders. Blood vessels are involved in diabetes, so its presence in this pattern is justified. However, prostate impairments constitute a non-cardiovascular chronic condition, so their inclusion in this pattern is more questionable. Older men mainly have this pattern, so gender may explain the mix of prostate and cardiovascular impairments in this pattern without any relationship between them other than shared risk factors.

We have observed a reduction in the proportion of anxiety and depression with increasing of age. The onset of physical illness common in older persons has been shown to increase proportion of depression and anxiety [35]. However, the literature showed that studies that have examined the incidence of anxiety or depression across the life span have inconclusive results. Often, anxiety or depression measures depend on cohort characteristics such as age, cultural background…[35]. Our sample is constituted of healthy voluntary participants who may represent a healthy cohort bias.

In our sample, cancer did not seem a component of multimorbidity patterns. Our sample was relatively young, with a low proportion of cancer.

The impact on HRQoL was greater with pattern A than B multimorbidity score. The greatest effects were found with the Duke Health Profile. A high pattern A score was associated with a lower score in both mental and physical dimensions of HRQoL and a high pattern B score was essentially associated with a lower score in the physical dimension. Indeed, no significant association was found between mental HRQoL score and pattern B score. Pattern B is related to cardiovascular and metabolic disorders. The literature showed that cardiovascular diseases were associated with a reduced in physical and mental dimensions of HRQoL [36]. In addition, a strong association was found between depression and cardiovascular diseases [37]. So we could have expected in our study a significant impact of pattern B multimorbidity score on mental HRQoL. In the absence of this result, we can make the hypothesis that our method allows us to better identify participants with cardiovascular pathology in absence of psychological disorders. In fact, anxiety/depression condition is represented in pattern A.

According to our analysis by age groups and gender, decrease in HRQoL was associated more with the DUKE than the SF-36, especially for pattern A score. This result was expected, considering that the Duke Health Profile has more items oriented toward mental health than the SF-36, which is a more general questionnaire. Several studies have shown that among all morbidities, mental disorders have the highest impact on HRQoL [10,38]. Nevertheless, other studies did not account for these conditions in HRQoL assessment [39]. Our results highlight the importance of considering mental disorders in HRQoL studies.

Many studies have shown decreased HRQoL with increasing number of chronic conditions [31,40,41]. With the methodology we used, we accounted for not only the number of morbidities but also their association in the population, which allowed for measuring the effect of morbidities on HRQoL as accurately as possible and comparing participants with each other by age group and gender.

Our approach allowed us to account for the interaction between chronic conditions and determine the multimorbidity status of each participant in contrast to studies that conducted a latent class analysis, seeking to consolidate clustered participants, which were unable to account for the complexity of the possibilities. This type of study incurs misclassification error and the model can be applied to only a limited number of participants [42]. When studies analyze morbidities individually, they can consider only a limited number of morbidity interactions [10]. Also it is not necessary to apply a method dedicated to multimorbidity for a specific population because our method allows for self-determination of a multimorbidity pattern.

Another strength of our study is the sample size. In addition, more than 5000 participants received a geriatric consultation. Nevertheless, our study could feature an underreporting of some diseases/conditions because of the self-administered questionnaires. Moreover, to assess the multimorbidity measure, we did not use a validated instrument such as the Cumulative Illness Rating Scale [43] or the Duke Severity Illness Checklist [44], both of which cannot be used for an existing cohort. Finally, we did not assess the severity of conditions and did not use an exhaustive list of conditions.

The results of our study of a relatively healthy sample, including a low prevalence of morbidities and high HRQoL, suggest that multimorbidity affects HRQoL differently depending on gender or age. Nonetheless, our study is a novel use of multimorbidity patterns to test the impact of multimorbidity on HRQoL. We found two patterns, which were clinically recognizable and theoretically plausible. Further investigations and research in older populations should consider multimorbidity patterns to confirm these findings.

## Conclusions and Future Research

Our analysis of more than 5,000 participants of 55 years and older, revealed two multimorbidity patterns which were clinically recognizable and theoretically plausible. These two identified patterns affected both HRQoL, notably a strong association was found between a multimorbidity pattern related to mental illness and deteriorated bone health (pattern A)–and a decrease in physical and mental HRQoL. The multimorbidity pattern related to cardiovascular and metabolic disorders (pattern B) seems to have no impact on mental HRQoL. The strength of theses associations differed according to age groups.

Our study is a new integrating approach of accounting for multimorbidity patterns in studying HRQoL in healthy population. Indeed, available multimorbidity indices were based on specific outcomes, such as mortality, costs, or function, and therefore may not address a patient’s overall condition [45]. In addition, they were validated on very specific populations and then it is difficult to apply them to other populations. The multimorbidity scores we identified (counted and weighted) can be used in clinical research to control for the effect of multimorbidity on patients’ HRQoL and may be useful for clinical practice.

To conclude, the results of this study could lead to a deeper understanding of the association of multimorbidity and HRQoL. Nevertheless, this method should be deepened through further studies to integrate the severity of conditions and to enrich the methodology.
